# The role of the Notch signalling pathway in the pathogenesis of ulcerative colitis: from the perspective of intestinal mucosal barrier

**DOI:** 10.3389/fmed.2023.1333531

**Published:** 2024-01-05

**Authors:** Hang Ning, Jiemin Liu, Jiaqian Tan, Mengni Yi, Xiaoyuan Lin

**Affiliations:** ^1^School of Traditional Chinese Medicine, Hunan University of Chinese Medicine, Changsha, China; ^2^Guizhou Provincial People’s Hospital, Guiyang, China; ^3^Medical School, Hunan University of Chinese Medicine, Changsha, China; ^4^The First Hospital of Hunan University of Chinese Medicine, Changsha, China

**Keywords:** ulcerative colitis, Notch, mucosal barrier, signalling pathway, review

## Abstract

Ulcerative colitis is a common digestive disorder worldwide, with increasing incidence in recent years. It is an urgent problem to be solved, as it seriously affects and threatens the health and life of the global population. Studies have shown that dysfunction of the intestinal mucosal barrier is a critical pathogenic factor and molecular basis of ulcerative colitis, and some scholars have described it as a “barrier organ disease.” While the Notch signalling pathway affects a series of cellular processes, including proliferation, differentiation, development, migration, and apoptosis. Therefore, it can regulate intestinal stem cells, CD4^+^ T cells, innate lymphoid cells, macrophages, and intestinal microbiota and intervene in the chemical, physical, immune, and biological mucosal barriers in cases of ulcerative colitis. The Notch signalling pathway associated with the pathogenesis of ulcerative colitis has distinct characteristics, with good regulatory effects on the mucosal barrier. However, research on ulcerative colitis has mainly focused on immune regulation, anti-inflammatory activity, and antioxidant stress; therefore, the study of the Notch signalling pathway suggests the possibility of understanding the pathogenesis of ulcerative colitis from another perspective. In this article we explore the role and mechanism of the Notch signalling pathway in the pathogenesis of ulcerative colitis from the perspective of the intestinal mucosal barrier to provide new targets and theoretical support for further research on the pathogenesis and clinical treatment of ulcerative colitis.

## 1 Introduction

Ulcerative colitis (UC) is a common gastrointestinal disorder characterised by continuous and diffuse inflammation of the colon mucosa. Due to the development in productivity and the improvements in living standards, people’s lifestyle and dietary habits have undergone significant changes, leading to a significant increase in the incidence of UC (1). The alternating clinical features of UC, including flare-ups, remissions, and relapses, increase the risk of cancer, reduce patients’ quality of life, and impose a significant burden on society (2). The mechanisms underlying the development of UC remain unclear; however, they may be associated with dietary habits (3), genetic susceptibility (4), environmental factors (5), autoimmune disorders (6), and mucosal barrier defects (7). Recently, extensive research has suggested that the abnormally high activity of the Notch signalling pathway may play a key role in the pathogenesis of UC (8). The Notch signalling pathway-mediated dysregulation of chemical, mechanical, immune, and biological barrier functions is considered a critical pathological mechanism of UC. Therefore, this study aimed to explore the role and mechanism of the Notch signalling pathway in the pathogenesis of UC from the perspective of the intestinal mucosal barrier to provide new targets and theoretical support for further research on the pathogenesis and clinical treatment of UC.

## 2 Notch signalling pathway composition and activation pathways

The Notch signalling pathway was initially discovered in fruit flies and was later found to be widely present in vertebrates and invertebrates. It is highly conserved in eukaryotes that transmit signals through cell–cell connections, influencing cellular processes such as proliferation, differentiation, development, migration, and apoptosis. It has a bidirectional regulatory role and plays a role in maintaining the homeostasis of tissues and organs. The Notch signalling pathway comprises Notch receptors and ligands, DNA-binding proteins [such as recombination signal binding protein-Jk (RBP-Jk)], regulatory factors, and downstream target genes [such as hairy and enhancer of split (Hes)1 and Math1]. In mammals, there are four types of Notch receptors (Notch1–4); Notch1 is primarily distributed in the intestine. There are five types of Notch ligands (Delta-like 1, 3, and 4, and Jagged 1 and 2).

The classical RBP-Jk-dependent activation pathway involves the following steps. The Notch precursor is transported to the Golgi apparatus. At the S1 site, it is cleaved by the Furin convertase, forming mature heterodimeric Notch receptors expressed on the cell membrane. When ligands bind to extracellular domains of Notch receptors, Notch signalling is activated. Subsequently, bound Notch receptors are endocytosed through hydrolysis at the S2 site by Metalloproteinases, the extracellular domain is released. Then the intracellular and transmembrane domain cleavage products are hydrolyzed at the S3 site by γ-secretase, releasing the active form of Notch, known as the Notch intracellular domain (NICD). The NICD is then translocated to the cell nucleus for signal transduction. It forms a trimeric transcription complex with RBP-Jk and recruits Mastermind-like transcriptional co-activators. This complex activates the transcription of downstream target genes of Notch, such as Hes and hairy/enhancer-of-split-related with YRPW motif, which are involved in cell fate determination. The activation pathways of the non-classical Notch signalling pathway may involve the activation of the Notch receptor by non-classical ligands, or activation in the absence of ligands, and this activation pathway is still to be further explored and studied (9).

## 3 Role of the Notch signalling pathway in the pathological process of UC

**Figure 1:**
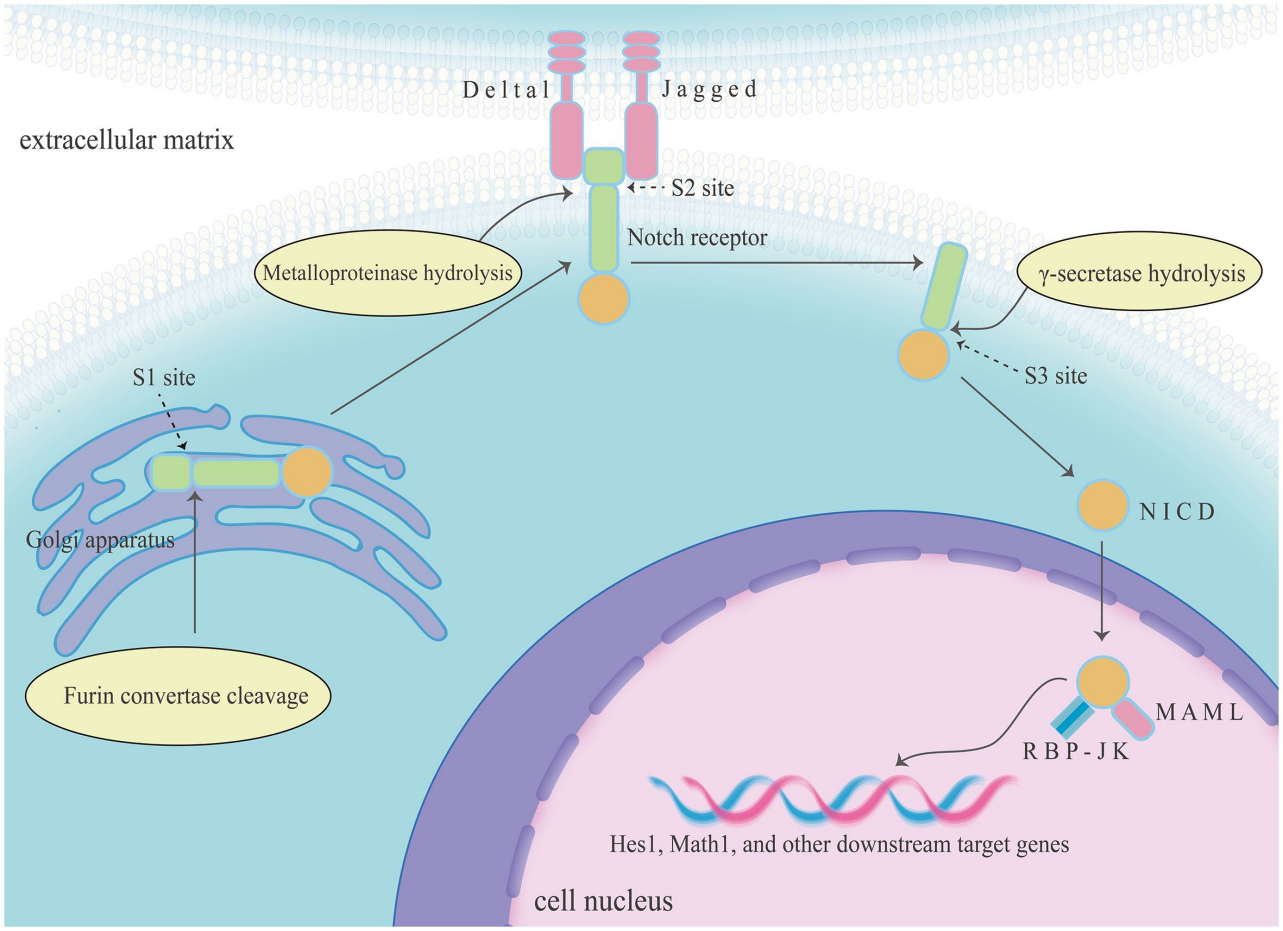
The Notch signalling pathway comprises Notch receptors and ligands, DNA-binding proteins (such as RBP-Jk), regulatory factors, and downstream target genes (such as Hes1 and Math1). The classical RBP-Jk-dependent activation of Notch signalling pathway including three key steps, and Notch receptors were turned into NICD in this process. Then NICD forms a trimeric transcription complex with RBP-Jk and recruits mastermind-like transcriptional co-activators in the cell nucleus, finally activates the transcription of downstream target genes of Notch.

The intestinal mucosal barrier is an important defence system in the intestine that effectively isolates harmful substances in the intestine to maintain barrier function and intestinal homeostasis. It is currently classified into four major categories: chemical, mechanical, immune, and biological barriers. The chemical barrier mainly includes the mucus, digestive juices secreted by the mucosal epithelium, and antimicrobial substances produced by the intestinal microbiota. The intestinal mucus is composed of mucin proteins, primarily mucin 2 (MUC2). The mechanical barrier includes the colonic epithelial cells and the connections between epithelial cells, including tight junctions (TJ), gap junctions, and adhesion junctions (AJ). The immune barrier mainly includes lymphoid tissue in the intestinal mucous membrane, which can protect the intestine from the invasion of pathogenic antigens through cellular and humoral immunity. The biological barrier is the normal symbiotic flora in the intestine, forming a mutually dependent microecosystem between the intestinal symbiotic flora and the host (10, 11). Numerous studies have shown that dysfunction of the intestinal mucosal barrier is a critical etiological factor and molecular basis of UC, and some scholars described it as a “barrier organ disease” (12). The Notch signalling pathway plays an important role in the mucosal barrier dysfunction (see Figure 1).

### 3.1 Chemical barrier

#### 3.1.1 Intestinal stem cell differentiation

**Figure 2:**
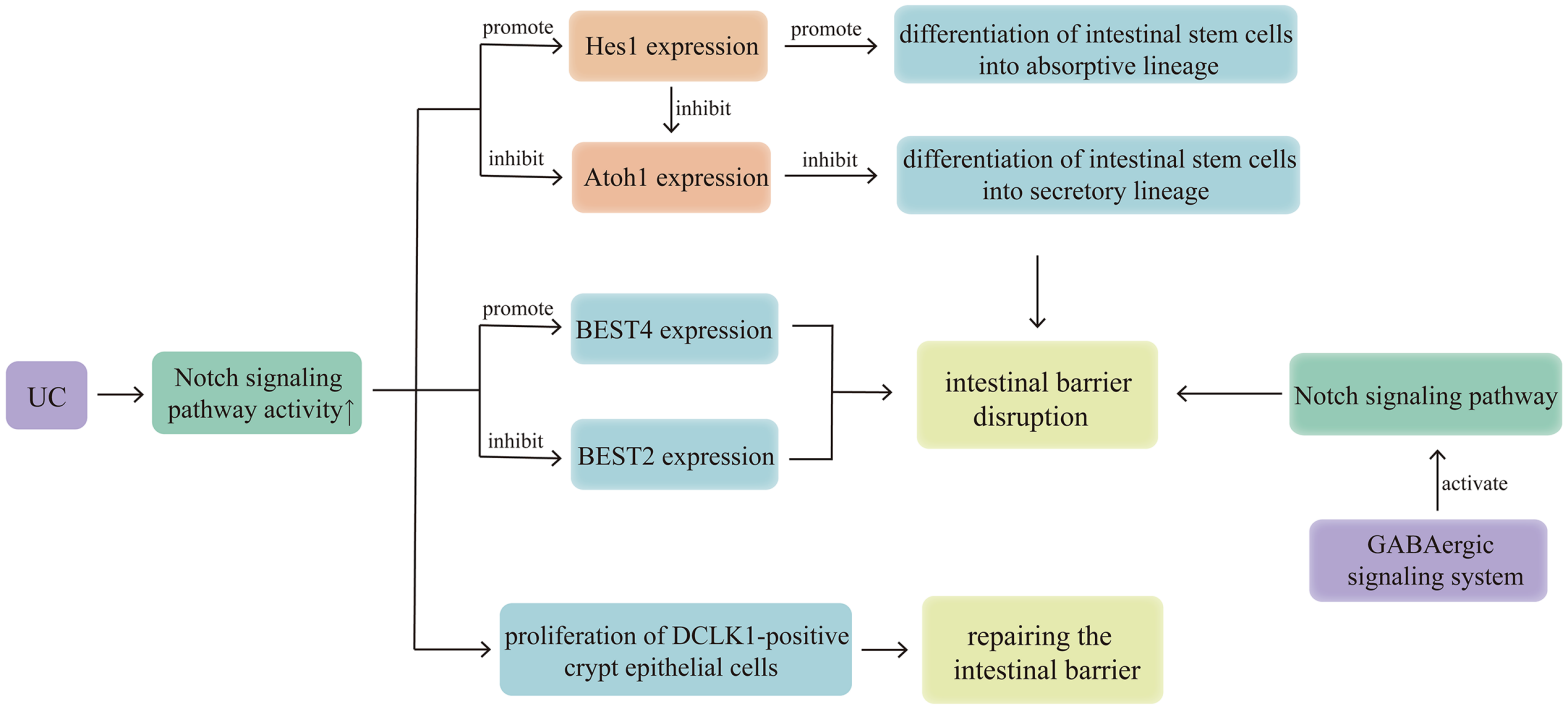
Notch signalling pathway regulates intestinal stem cell differentiation, BESTs, DCLK1 and GABA to affect the development of UC. Activation of the GABAergic signalling system in the colonic epithelium can activate the Notch signalling pathway, thereby disrupting the intestinal epithelial mucosal barrier, this suggests that the brain-gut axis may mediate the involvement of the Notch signalling pathway in UC pathogenesis. In cases of UC, the Notch signalling pathway exhibits abnormally high activity, inhibiting differentiation into the secretory lineage, down regulating the expression of BEST2, up regulating the expression of BEST4, finally weakening the mucous barrier. And there is a self-protection mechanism, activation of the Notch signalling pathway can promote the expression of DCLK1, benefit to crypt repair and suppression of inflammation in patients with UC.

Under normal conditions, the Notch signalling pathway maintains a relatively stable balance between the differentiation of intestinal stem cells into secretory and absorptive lineages. However, in cases of UC, the Notch signalling pathway exhibits abnormally high activity, inhibiting differentiation into the secretory lineage. And the secretory cells are the main producers of MUC2, so inhibiting secretory lineage differentiation ultimately leads to weakening of the mucous barrier (10). The basic helix-loop-helix transcription factors, Hes1 and atonal homolog 1 (Atoh1), are downstream target genes of Notch. Hes1 expression inhibits Hath1 (the human homolog of Atoh1), promoting the differentiation of intestinal stem cells into the absorptive lineage (13). High Atoh1 activity promotes the differentiation of intestinal stem cells into the secretory lineage (14). Increased Notch signalling activity increases Hes1 expression and inhibits Atoh1 activity. In addition, the sterile alpha motif pointed domain-containing ETS transcription factor has been identified as a transcriptional co-regulator of Atoh1 that can amplify Atoh1-dependent transcription but cannot independently initiate transcription of Atoh1 target genes (Figure 2) (15).

#### 3.1.2 Bestrophins

Bestrophins (BESTs) are newly discovered genes that encode ion channels and can function as chloride (Cl^−^), bicarbonate (HCO_3_^−^), or voltage-gated calcium channels (16). Notably, four members of the BEST family (BEST1–4) have been identified in humans (17). Experimental studies have shown that BEST2 and 4 are expressed in a lineage-specific manner in the human intestinal epithelium, with BEST2 being expressed only in colonic goblet cells and BEST4 being expressed in absorptive cells of the small intestine and colon. Upregulation or downregulation of Notch signalling pathway activity leads to preferential expression of BEST4 or 2, respectively, in the cells (18). Previous studies have suggested that the significant downregulation of ion channels [such as sodium–hydrogen exchanger (NHE) 3 and Cl/HCO_3_ exchanger down-regulated in adenoma] in UC active lesions (19) is one of the molecular basis for severe diarrhoea (20). Since BEST2 gene knockout mice spontaneously develop colitis (21), the mechanism of action of BEST2 in cases of UC may be similar to that of other ion channels (such as NHE1 and NHE3) (22, 23), with high activation of the Notch signalling pathway inhibiting the expression of BEST2, which may be one of the mechanisms underlying UC pathogenesis (Figure 2).

#### 3.1.3 Doublecortin-like kinase 1

Doublecortin-like kinase 1 (DCLK1) is a marker of intestinal tuft cells and can promote intestinal repair to alleviate mucosal barrier dysfunction (24). Inhibiting Notch signalling reduces DCLK1-positive crypt epithelial cells after radiation injury (25). The inhibition of DCLK1-positive crypt epithelial cell proliferation caused by blocking Notch signalling is associated with microbial dysbiosis, mucosal barrier disruption, and release of pro-inflammatory mediators, suggesting that the Notch signalling pathway is a key intermediate pathway regulating DCLK1 expression, and the modulation of Notch signalling is important for crypt repair and suppression of inflammation in patients with UC (Figure 2) (26).

#### 3.1.4 γ-aminobutyric acid

Gamma-aminobutyric acid (GABA) is the major inhibitory neurotransmitter in the central nervous system and is mainly distributed in the brain and spinal cord. GABA, GABA receptors, and their metabolic enzymes are collectively referred to as the GABAergic signalling system. Activation of the GABAergic signalling system in the colonic epithelium can inhibit the transformation of colonic crypt stem cells into goblet cells and reduce epithelial cell proliferation by activating the Notch signalling pathway, thereby disrupting the intestinal epithelial mucosal barrier (27). This suggests that the brain-gut axis may mediate the involvement of the Notch signalling pathway in UC pathogenesis (Figure 2).

#### 3.1.5 Olfactomedin-4

**Figure 3:**
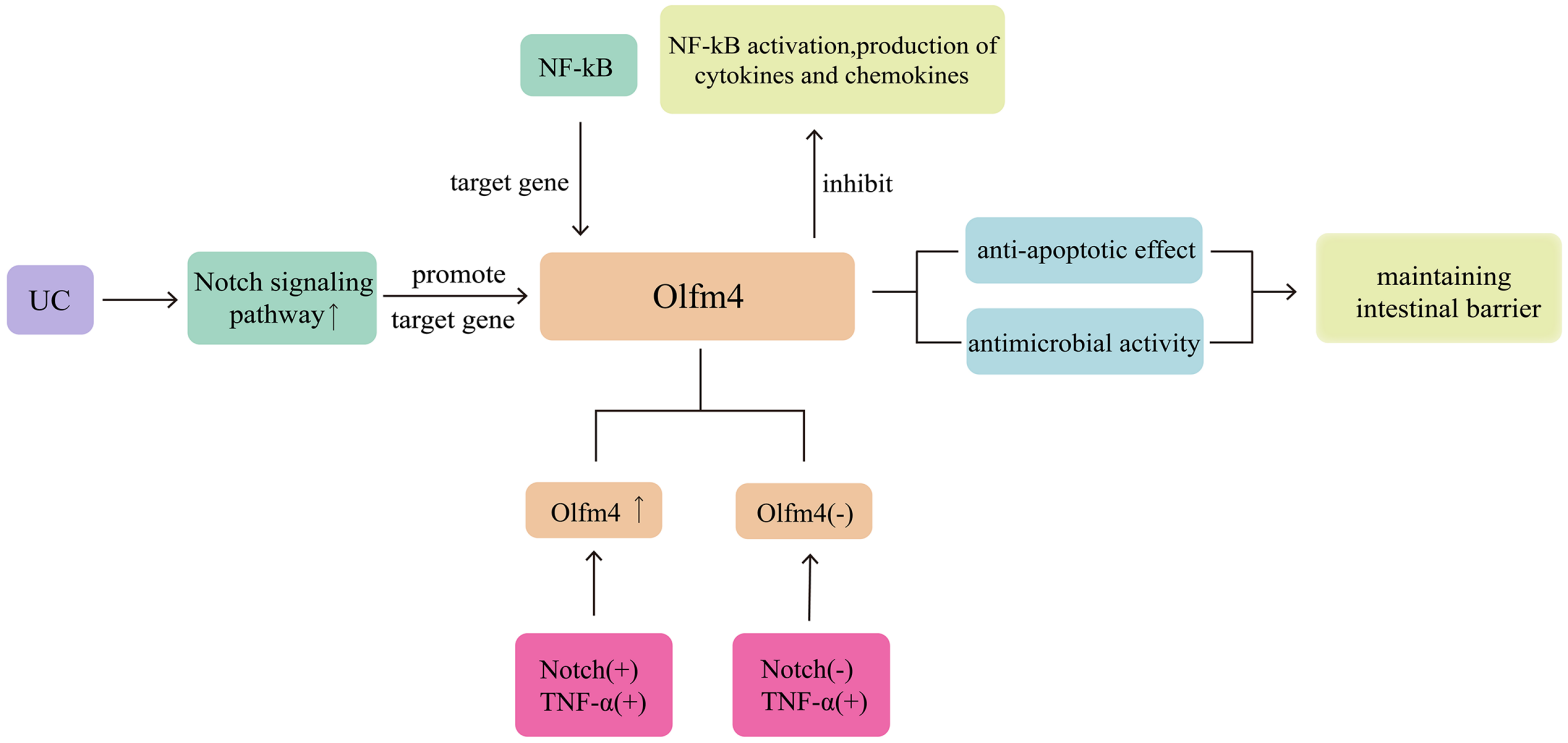
Olfm4 exhibit antimicrobial activity and show cell-protective effect against apoptosis. Activation of the Notch signalling pathway can promote the expression of Olfm4. At the same time, it is also showing anti-inflammatory activity and can inhibit NF-κB activation.

Olfactomedin-4 (Olfm4) is a glycoprotein belonging to the olfactomedin family and is considered a marker for intestinal stem cells. It is highly expressed in the small intestine and colon, expressed in the stomach and bone marrow, and weakly expressed or not expressed in other tissues (28). Secreted Olfm4 can bind to defensins and exhibit antimicrobial activity (29). Cytoplasmic Olfm4 has a cell-protective effect against apoptosis in colonic epithelial cells of patients with inflammatory bowel disease (IBD) (30). Olfm4 expression is significantly increased in inflamed tissues of patients with UC, extending to the surface of epithelial cells and crypts; however, under normal conditions, Olfm4 expression is limited to the lower one-third of the crypts in normal tissues (29). Olfm4 is a target gene of nuclear factor kappa-B (NF-κB) and can also bind to Nucleotide oligomerisation domain (NOD) 1 and 2 to inhibit NF-κB activation and the production of cytokines and chemokines (31). In addition, Olfm4 is a target gene of the Notch signalling pathway, and its transcription depends on the activation of the RBP-Jk binding site. Inhibition of Notch1 suppresses Olfm4 expression, inhibiting the proliferation of intestinal stem cells (32). Treating the intestines with Jagged1 after lipopolysaccharide (LPS) stimulation upregulates the expression of Notch1 and Olfm4, reducing cell apoptosis and the production of reactive oxygen species (ROS) (33). Furthermore, incubating cells with tumour necrosis factor-alpha (TNF-α) alone does not affect Olfm4 expression (29); however, the combined use of TNF-α and components of the Notch signalling pathway can upregulate Olfm4 expression (30). TNF-α is one of the most important pro-inflammatory cytokines promoting IBD, further highlighting the role of Olfm4 in intestinal inflammation (Figure 3).

#### 3.1.6 Wnt/β-catenin signalling pathway

Intestinal stem cells expressing leucine-rich repeat-containing G-protein coupled receptor 5 (LGR5^+^) exhibit self-renewal and high differentiation potentials, playing an important role in the repair of intestinal mucosal damage. The Wnt/β-catenin and Notch signalling pathways collectively maintain the function of intestinal stem cells expressing LGR5^+^ (34). Both pathways exhibit opposing activities in regulating intestinal stem cell differentiation. The Wnt signalling pathway promotes intestinal stem cell differentiation into the secretory lineage, whereas the Notch signalling pathway promotes intestinal stem cell differentiation into the absorptive lineage, resulting in a reduction in goblet cells (35). Therefore, the interaction between the Wnt/β-catenin and Notch signalling pathways is considered to influence intestinal stem cell differentiation and mucosal barrier repair (36). Notably, the disruption of the Notch/Wnt signalling pathways can lead to mucosal barrier disruption and intestinal inflammation (37). The mechanisms of mutual interference between the Notch and Wnt signalling pathways have also been preliminarily elucidated. Among these mechanisms, the rapid switching between Wnt-ON/Notch-OFF and Wnt-OFF/Notch-ON states provides a solid molecular basis for Notch-targeting Wnt signal transduction. The main pathway for Wnt-ON/Notch-OFF is mediated by Wnt-related proteins, Dishveled, which inhibits Notch and RBP-Jk, and GSK3, which physically binds and phosphorylates Notch intracellular domain (NICD) 1 and 2 (35). Wnt-OFF/Notch-ON may be associated with Notch-mediated activation of β-catenin by Numb, interfering with β-catenin’s endolysosomal transport and degradation and preventing β-catenin accumulation (38).

**Figure 4:**
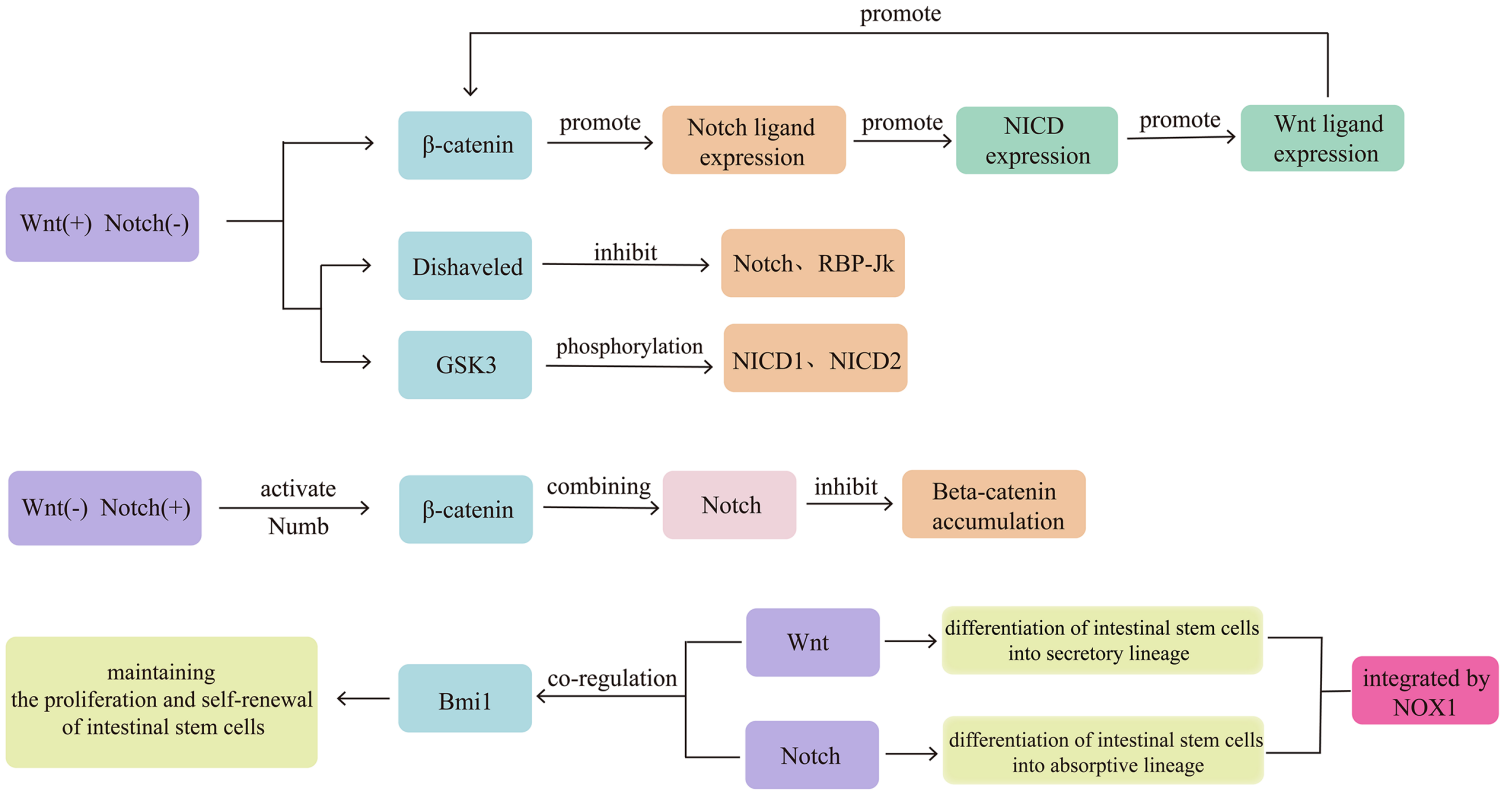
Intestinal stem cells expressing LGR5^+^ exhibit self-renewal and high differentiation potentials, playing an important role in the repair of intestinal mucosal damage. The Wnt/β-catenin and Notch signalling pathways collectively maintain the function of intestinal stem cells expressing LGR5^+^. Both pathways exhibit opposing activities in regulating intestinal stem cell differentiation. Therefore, the interaction between the Wnt/β-catenin and Notch signalling pathways is considered to influence intestinal stem cell differentiation and mucosal barrier repair. The signals of Wnt/β-catenin and Notch1 can be integrated by NOX1.

In addition, the Notch and Wnt signalling pathways can interact with each other to initiate sequential signal transduction. The typical Wnt signal transduction in intestinal progenitor cells can activate the transcription of the Jagged1 gene, leading to corresponding Notch signal pathway transduction and activation of corresponding Notch target gene transcription (39). Notch signal transduction in macrophages (Mø) can enhance the expression of Wnt ligands (40). Furthermore, nicotinamide adenine dinucleotide phosphate oxidase 1, which produces ROS and is highly expressed in colonic epithelial cells, can integrate Wnt/β-catenin and Notch1 signals, potentially serving as an important molecular mechanism for the interaction between the Wnt/β-catenin and Notch1 signalling pathways to control colonic cell proliferation and differentiation direction (41). B lymphoma Moloney murine leukemia virus insertion region 1 homolog, a transcriptional repressor belonging to the Polycomb family, is considered to be jointly regulated by the Wnt/β-catenin and Notch signalling pathways, contributing to the maintenance of intestinal stem cell proliferation and self-renewal (Figure 4) (42).

### 3.2 Mechanical barrier

**Figure 5:**
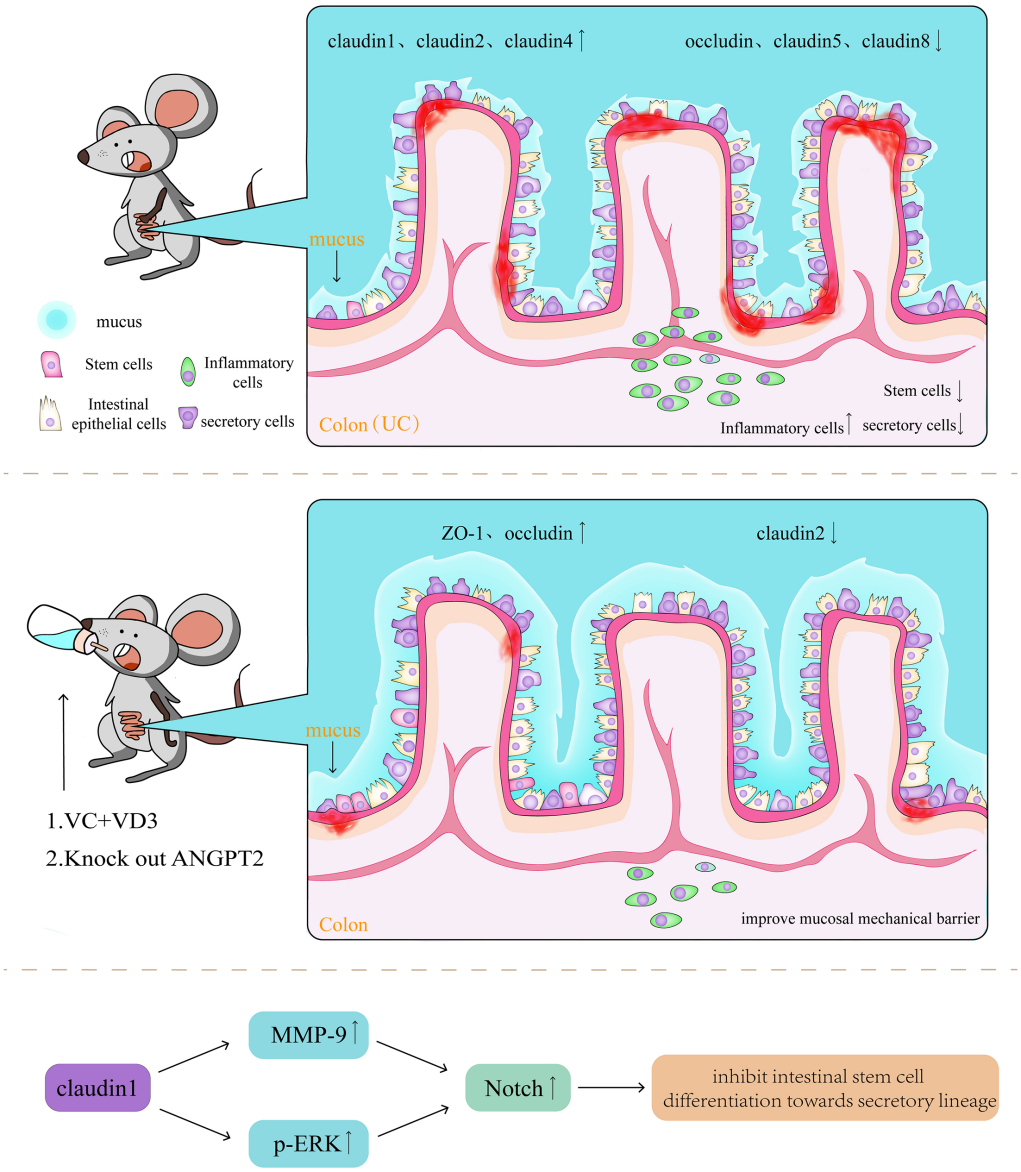
In active UC intestines, both TJ and AJ proteins exhibit pathological changes, in expression, distribution, and alignment. Claudin1, 2, and 4 are upregulated, whereas occludin, claudin5, and claudin8 are downregulated. Blocking the Notch signalling pathway, can improve the barrier function of the mucosal mechanical barrier by upregulating the expression of Zonula occludens-1 and occludin and downregulating claudin2 expression.

Below the intestine’s mucus layer, intestinal epithelial cells are connected through TJ and AJ, forming the mechanical barrier of the intestinal mucosa. However, in active UC intestines, both TJ and AJ proteins exhibit pathological changes, in expression, distribution, and alignment. Notably, claudin1, 2, and 4 are upregulated, whereas occludin, claudin5, and claudin8 are downregulated. Furthermore, the expression levels of claudin1 and 2 are positively correlated with inflammatory activity (43–45). Experimental studies have found that blocking the Notch signalling pathway through the knockout of angiopoietin-2 (46) or using a combination of vitamin C and D3 (47) can improve the barrier function of the mucosal mechanical barrier by upregulating the expression of Zonula occludens-1 and occludin and downregulating claudin2 expression. The Notch signalling pathway mediates cell communication in various tissues and at different stages of organism development. Its activation depends on the interaction between transmembrane ligands and neighbouring cell receptors. Therefore, studies have also reported the regulatory role of TJ and AJ in Notch signalling (48). Notably, claudin1 expression can induce Matrix metallopeptidase 9 and phosphorylation of extracellular signal-regulated protein kinase signalling to activate Notch signalling, thereby inhibiting the differentiation of intestinal stem cells into secretory lineages. It can also induce colonic epithelial proliferation in a Notch-dependent manner (49). This mechanism is also associated with the pathogenesis of colitis-associated colorectal cancer (CAC) (Figure 5) (50).

### 3.3 Immune barrier

#### 3.3.1 CD4^+^ T cell

CD4^+^ T cells are key components of the immune barrier and inflammatory response. When the balance between them is disrupted, CD4^+^ T cells are considered a major driving factor in cases of IBD, with major subsets associated with UC, including T helper (Th) 17, 1, and 2 (51). The Notch1 signalling pathway regulates the generation and migration of gamma delta T cells, the maturation of alpha beta T cells, the differentiation of CD4^+^ T cells into Th1 and 2, and the activation of T cells (52), thus indicating the importance of the Notch signalling pathway in CD4^+^ T cells. Identifying the mechanisms through which the Notch signalling pathway affects CD4^+^ T cell differentiation will help us find new targets for IBD treatment.

Studies have generally supported the role of the Notch1 signalling pathway as a regulator of Th17/regulatory T cell balance (53); however, there is still no consensus on its exact mechanism. Furthermore, the Notch signalling pathway promotes the Th17 function. High Delta-like ligand 4 expression can enhance the promoting Th17 differentiation effects of dendritic cells (DCs) (54), and upregulating the expression of retinoic acid-related orphan receptor gamma (RORγ) in CD4^+^ T cells promotes Th17 differentiation (55). RBP-Jk is a key driver of interleukin (IL)-23R expression and inhibits the production of anti-inflammatory IL-10 by Th17 cells, regulating the development of pathogenic and non-pathogenic Th17 cells (56). It also exhibits negative regulation of Th17. Loss of Notch1 in CD4^+^ T cells in the gut and gut-associated lymphoid tissues (GALTs) (57) and loss of RBP-Jk in DCs (53) enhance Th17 differentiation. Treatment with Jagged1, which leads to high transmission of Notch1 signals, can inhibit the expression of the Th17 transcription factor RORγt, thereby suppressing Th17 differentiation (58). The Notch1 signalling pathway also contains a long intergenic non-coding RNA (lincRNA) called XLOC_00026, where XLOC_000261 is a negative regulator of RORγt. Knockout of lincRNA XLOC_000261 leads to a significant increase in RORγt expression (59). Current research has confirmed the regulatory role of the Notch signalling pathway in T cell differentiation, and future research needs to clarify how different Notch ligands and receptors interact with each other and control different responses to regulate T cells.

Th1 and 2 have their specific roles, with Delta inducing Th1 and Jagged inducing Th2. Toll-like receptors (TLR)4-induced DC high expression of Delta-like ligand 4 can promote T-box expressed in T cells (T-bet)-dependent Th1 differentiation (60) and regulate Th1 production of IL-10 through a signal transducer and activator of transcription (STAT)4-dependent process, transforming pro-inflammatory Th1 cells into T cells with regulatory activity (61). In addition, TLR signalling can strongly suppress Jagged2 expression, thereby interfering with Th1 and 2 differentiation (62). Jagged can induce GATA Binding Protein 3 (GATA3) and directly regulate IL-4 gene transcription through the RBP-Jk site on the 3′ enhancer, independent of IL-4/STAT6 guidance for Th2 differentiation (63). In addition, the Notch1 signalling pathway promotes CD4^+^ T cell differentiation towards Th1 by inhibiting microRNA (miR)-29 and relieving its transcriptional repression of the pro-inflammatory genes, T-Box Transcription Factor 21 and interferon-gamma (IFN-γ) (64). Fever is a prevalent symptom of the inflammatory response, and in cases of moderate fever (39°C), alterations in CD4^+^ T cells biased to Th2 differentiation regulated by Transient receptor potential type v 1 are mediated by the Notch1 signalling pathway (65). These findings suggest that the intervention of Notch ligands in Th1 or 2 differentiation cannot occur independently of cytokines and cannot reset cytokine-driven Th1 or 2 differentiation. This implies that cytokines mediate the influence of the Notch signalling pathway on Th1 and 2 differentiation. Therefore, it is necessary to clarify how Notch receptors initiate different transcription profiles based on different ligands.

**Figure 6:**
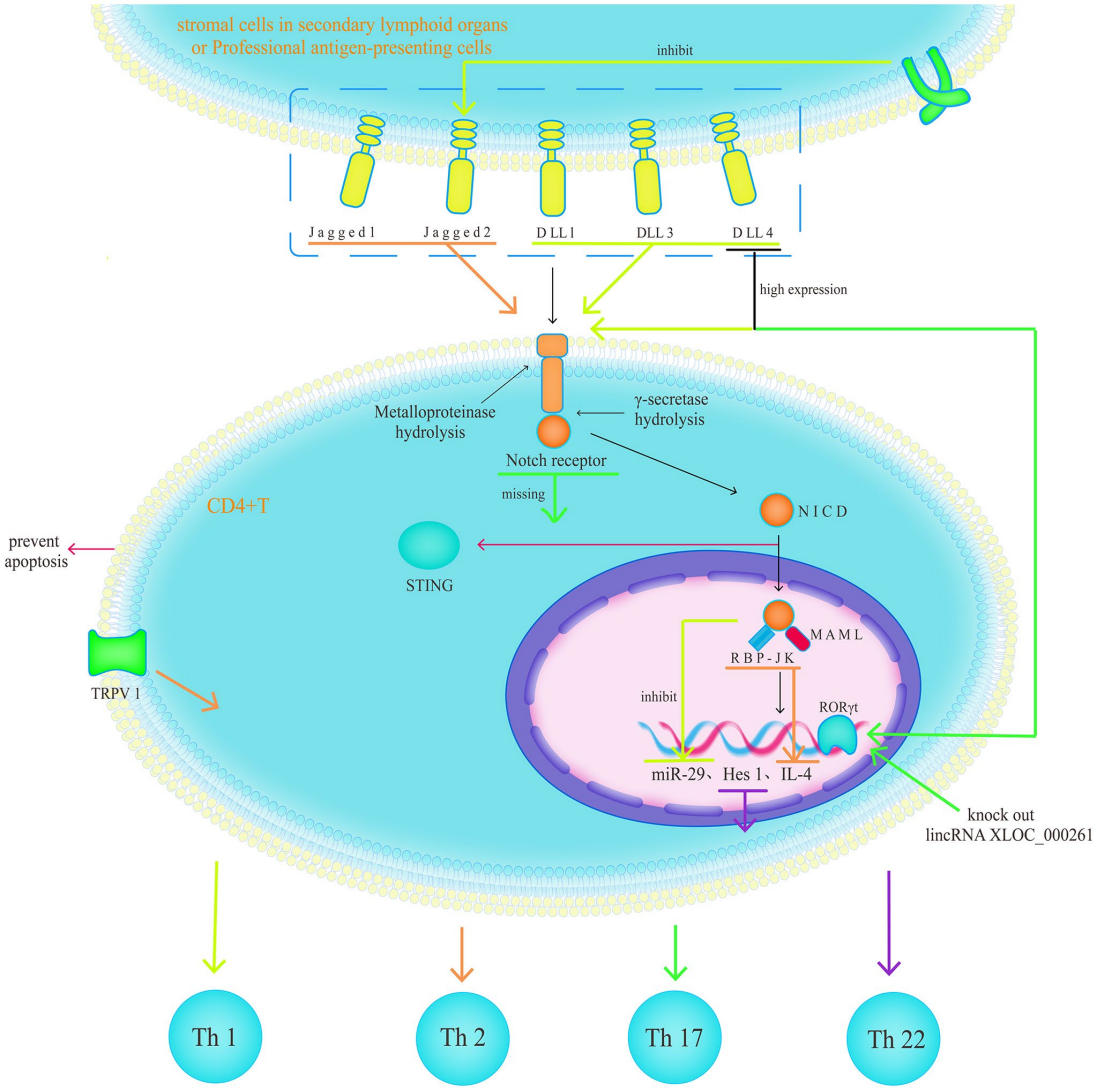
The Notch1 signalling pathway as a regulator of Th17/regulatory T cell balance; however, there is still no consensus on its exact mechanism. Th1 and 2 have their specific roles, with Delta inducing Th1 and Jagged inducing Th2. The intervention of Notch ligands in Th1 or 2 differentiation cannot occur independently of cytokines and cannot reset cytokine-driven Th1 or 2 differentiation.

Activation of the Stimulator of Interferon Genes (STING) induces apoptosis in T cells, and NICD can interact with STING in the Cyclic dinucleotide (CDN) binding domain and compete with CDN to inhibit STING activation, thus protecting CD4^+^ T cells from STING-mediated cell apoptosis (66). Hes1 has been suggested as a key promoting factor for CD4^+^ T cell differentiation towards Th22, which is crucial for mucosal immunity (67). Finally, matrix cells beneath the Follicle-associated epithelium highly express Delta-like ligand 1 to maintain Notch1 signalling, thereby limiting lineage differentiation and promoting the complete maturation of GALTs (Figure 6) (68).

#### 3.3.2 Innate lymphoid cells

The innate lymphoid cells (ILC) family can be divided into three subgroups based on their transcription factors and cytokine production profiles: type 1 ILCs (including natural killer cells and ILC1), type 2 ILCs (ILC2), and type 3 ILCs [including lymphoid tissue inducer cells (LTis) and ILC3], which are considered innate counterparts of Th1, 2, and 17/22 cells, respectively. The discovery and functional studies of ILCs have greatly enhanced the understanding of immune regulation in mucosal tissues, particularly the important role of ILC3 in intestinal immune responses and tissue repair (69). The development and lineage classification of ILCs remain controversial; however, existing research suggests that the Notch signalling pathway plays a crucial role in these processes. Notch signalling is essential for the silencing of the early B cell factor 1 and paired box 5 in common lymphoid progenitor cells (70). Subsequently, Notch signalling acts in parallel with GATA3 to form a population of ILC/T progenitor cells (71). Then, the inhibitor of DNA binding 2 (Id2) plays a role in transcriptional repression, inhibiting the differentiation towards T cells by suppressing the expression of E-box family transcription factors, thereby promoting the differentiation of ILC/T progenitor cells towards common helper-like innate lymphoid progenitors (ChILPs) (72). ChILPs can be divided into Promyelocytic leukaemia zinc finger (PLZF) + ChILPs and PLZF − ChILPs based on the expression of PLZF, with PLZF + ChILPs developing into PLZF − ChILPs under Notch signalling (73). PLZF − ChILPs can differentiate into LTis under the influence of Notch1, Aryl Hydrocarbon Receptor (AHR) (74), RORγt (75), and Thymocyte selection-associated high-mobility group box protein (76). ILC3 can be divided into different subgroups based on the expression of natural cytotoxicity receptors (NCRs) on their surface. PLZF + ChILPs can differentiate into NCR − ILC3 under the influence of AHR (74), Notch1 (77), and RORγt (78). The mechanisms associated with the Notch1 signalling pathway and the RORγt are related to the promotion of the expression of IL-7R and IL-23R receptors in PLZF + ChILPs. NCR − ILC3 can differentiate into NCR + ILC3 under the influence of Notch, T-bet (79), and T cell factor 1 (80), and the maintenance of NCR + ILC3 also requires continuous Notch signalling (81).

**Figure 7:**
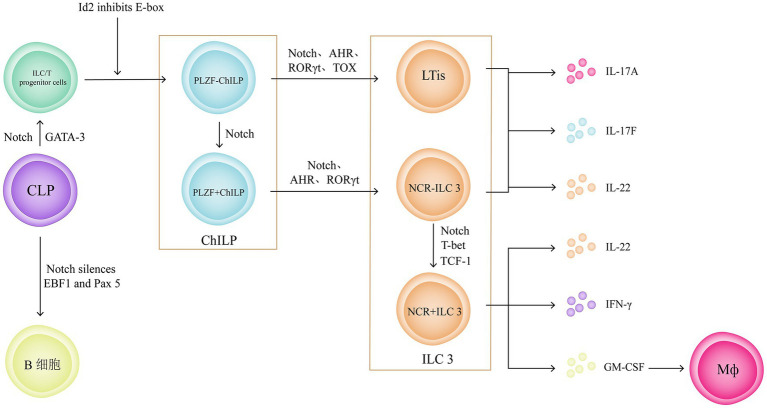
ILC3 plays an important role in the pathogenesis of UC. And the Notch signalling pathway plays a crucial role in development and lineage classification of ILCs. Notch signalling can silencing the early B cell factor 1 and paired box 5 in common lymphoid progenitor cell to form a population of ILC/T progenitor cells. Then, inhibiting the differentiation towards T cells by suppressing the expression of E-box family transcription factors, thereby promoting the differentiation of ILC/T progenitor cells towards ChILPs. ChILPs can be divided into Promyelocytic leukaemia zinc finger (PLZF) + ChILPs and PLZF − ChILPs. PLZF + ChILPs can differentiate into NCR − ILC3 under the influence of Notch signalling pathway.

Experimental results (82) and clinical applications (83) support the conclusion that ILC3 plays an important role in the pathogenesis of UC. LTis and NCR − ILC3 mainly produce IL-17A, IL-17F, and IL-22, whereas NCR + ILC3 mainly produces IL-22 and IFN-γ (84). IL-22 and IFN-γ play important roles in regulating intestinal mucosal immunity, maintaining intestinal microbiota homeostasis, and promoting mucosal tissue repair (69). IL-17 produced by LTis and NCR − ILC3 accumulates in the intestines of patients with UC (85), and the abundant secretion of Granulocyte-macrophage colony-stimulating factor (GM-CSF) by NCR + ILC3 induces the accumulation of monocyte-derived Mø, exacerbating intestinal inflammation (Figure 7) (86).

#### 3.3.3 Macrophages

Mø are essential components of the intestinal immune system and are closely associated with the pathogenesis of UC. They have also been proven to be reliable targets for UC treatment (87). Mø can be divided into resident and inflammatory Mø, and based on their roles in inflammatory responses, they can be further classified as pro-inflammatory M1 and anti-inflammatory M2 (88). In inflammation, resident Mø express TLRs but do not respond to TLR ligand stimulation. They do not release pro-inflammatory cytokines but retain strong phagocytic and bactericidal activities (89). They secrete IL-4, IL-10, mannose receptor C type 1, Cluster of Differentiation 163, arginase-1, IL-1 receptor antagonist, and stabilin-1, preventing excessive inflammation and promoting tissue healing, and are considered to be M2 Mø. Furthermore, monocytes in the blood are recruited to the site of inflammation and respond to TLR ligand stimulation, forming inflammatory Mø (90), which secrete IL-1, IL-6, TNF-α, IL-12, and IL-23 and produce ROS, reactive nitrogen intermediates, and proteases. They induce inflammation and clear pathogens and are considered to be M1 Mø. Therefore, regulating the balance between M1 and M2 is of great significance for UC treatment.

In patients with UC, increased M1 and decreased M2 polarisation occur with high transmission of the Notch1 signalling pathway. Inhibiting Notch1 significantly reduces M1 polarisation, increases M2 polarisation and anti-inflammatory markers, and restores the M1/M2 balance (91). This phenomenon is partially mediated by miR148a-3p, which is a downstream molecule of the Notch1 signalling pathway. Its direct target gene in Mø is Phosphatase and tensin homolog (PTEN). Therefore, Notch1 signalling mediates the PTEN/Protein kinase B (AKT) pathway to increase ROS production, enhance the phagocytic and bactericidal capabilities of Mø, and upregulate NF-κB to enhance M1 polarisation and pro-inflammatory response (92). Notch1 signalling activated by Delta-like ligand 4 inhibits IL-4/IL-4R signalling, thereby inhibiting the Janus kinase (JAK)/STAT pathway and suppressing M2 polarisation. It also drives early and late apoptosis of IL-4- and IL-10-induced M2, respectively (93). Signal-regulatory protein (SIRP) α promotes Mø polarisation towards M2 and inhibits their phagocytic function. The Notch1 signalling pathway directly downregulates the expression of SIRPα through negative regulation by Hes1, thereby inhibiting the phosphorylation of Src homology 2 domain-containing phosphatase-1 and 2 and increasing M1 polarisation (94, 95).

Notably, the Notch1 signalling pathway interacts with other signalling pathways. First, TLR signalling in Mø upregulates the expression of Jagged1 in an RBP-Jk-dependent manner, enhancing Notch1 signalling (96). TLR2-mediated Notch1 upregulation is completely dependent on Myeloid differentiation primary response protein 88 (MyD88) (97). Second, Notch1 promotes NF-κB nuclear translocation and DNA binding, enhancing LPS-induced TLR4-triggered NF-κB signalling through increased expression of TNF receptor-associated factor 6 (TRAF6), phosphorylation and activation of IkappaB (IκB) kinase (IKK)-α/β, and degradation of IκBα, indicating the cylindromatosis-TRAF6-IKK pathway (98, 99). IFN regulatory factors (IRFs) are transcriptional regulators of Mø polarisation and are considered downstream targets of the Notch1/RBP-Jk pathway. RBP-J promotes the synthesis of IRF8 protein by selectively augmenting kinase IL-1 receptor-associated kinase-like 2-dependent signalling through TLR4 to the kinase mitogen-activated protein kinase-interacting kinase 1 and downstream translation-initiation control through Eukaryotic translation initiation factor 4E, integrating the Notch1/RBP-Jk and TLR4 signalling pathways at the level of IRF8 protein synthesis, thereby regulating M1 polarisation (100). RBP-Jk is considered a strong transcriptional repressor of NF-κB, which is overcome by activated Notch1 (101). It can also increase the expression of Monocyte to macrophage differentiation-related (MMD) in LPS-induced Mø. Overexpression of MMD enhances phosphorylation of ERK1/2 and Akt, leading to increased TNF-α and nitric oxide production (102). Activation of the Notch1 signalling pathway in Mø can directly regulate c-Rel and indirectly regulate TNF-α to affect IL-12 expression (103). The NF-κB transcriptional inhibitory factor IκB binds to the promoter region of the Notch target gene Hes1, and TNF-α stimulation can release IκB, promoting NF-κB synthesis (104). In addition, the Notch1 signalling pathway regulates the expression of GM-CSF through the Jun N-terminal kinase/NF-kB signalling pathway, participating in the regulation of Mø apoptosis (105).

**Figure 8:**
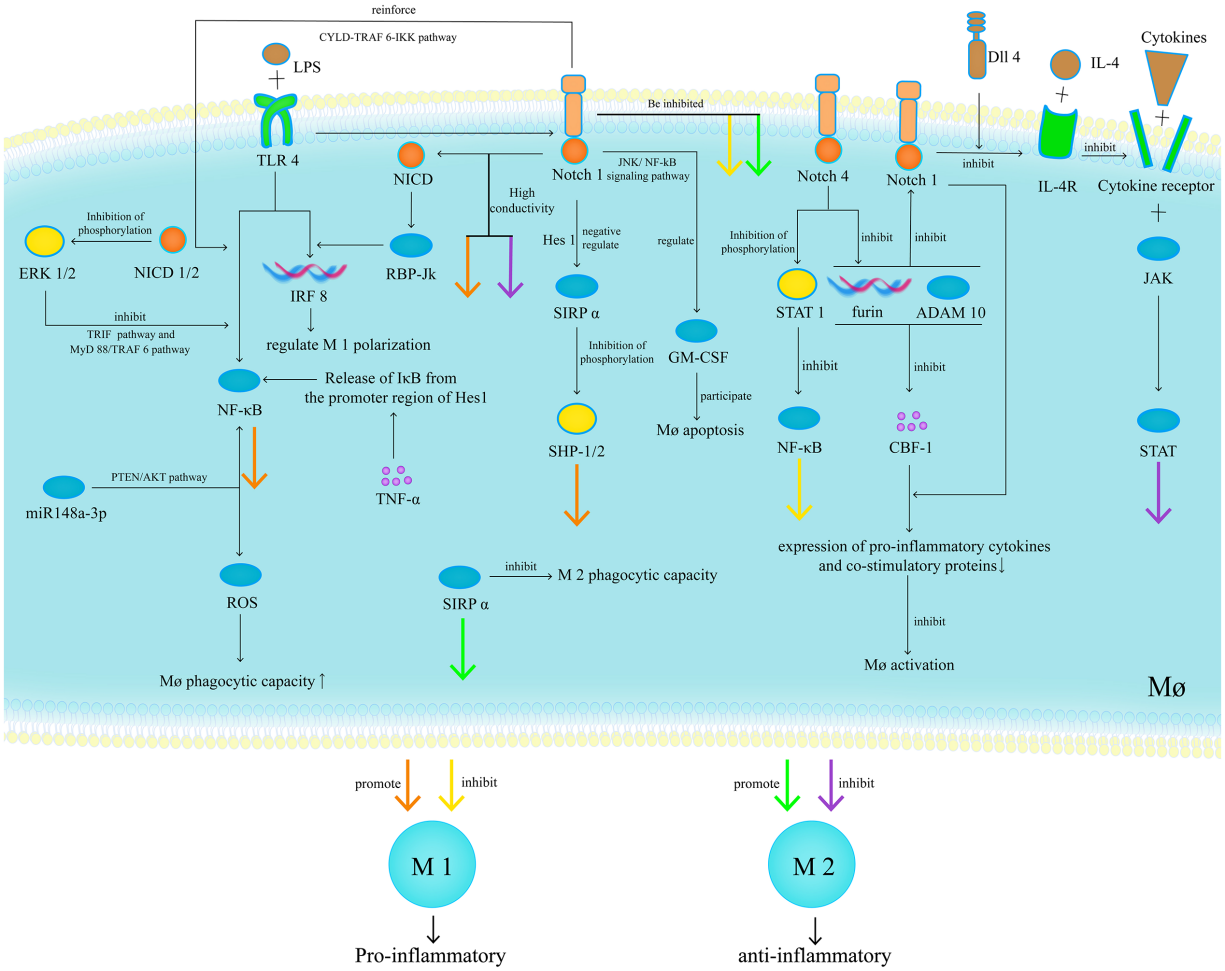
In patients with UC, increased M1 and decreased M2 polarisation occur with high transmission of the Notch1 signalling pathway. Inhibiting Notch1 significantly reduces M1 polarisation, increases M2 polarisation and anti-inflammatory markers, and restores the M1/M2 balance. Furthermore, Notch signalling exhibits partial anti-inflammatory effects.

Furthermore, Notch signalling exhibits partial anti-inflammatory effects. Delta-like ligand 1 can inhibit monocyte-to-Mø differentiation (106). NICD1 and 2 inhibit ERK1/2 phosphorylation through their PEST domain, thereby inhibiting TLR-induced NF-κB transcriptional activity through the MyD88/TRAF6 and Toll/IL-1 receptor domain-containing adapter protein pathways, ultimately reducing the production of pro-inflammatory factors in activated Mø (107). Furthermore, Notch4 exhibits distinct anti-inflammatory properties compared with other Notch receptors. It inhibits NF-κB activity by inhibiting STAT1 phosphorylation and activation. It also inhibits the activity of RBP-Jk and three other Notch receptors by inhibiting furin expression and A Disintegrin and metalloproteinase domain-containing protein 10 activation, ultimately reducing the expression of pro-inflammatory cytokines and co-stimulatory proteins and exerting a negative regulatory effect on Mø activation (108). Finally, the E-Cad-mediated adhesion between Mø and intestinal epithelial cells is associated with the activation of Notch signalling in epithelial cells. Excessive adhesion disrupts normal epithelial differentiation and promotes goblet cell depletion, leading to ecological imbalance and intestinal inflammation (Figure 8) (109).

### 3.4 Biological barriers

The gut microbiota is considered an important factor in the pathogenesis of IBD, especially when it is unclear whether the gut microbiota is a major or minor factor. The interaction between the Notch signalling pathway and gut microbiota is an important research direction for regulating host life activities and immune responses. Current research on this interaction mainly focuses on the influence of gut microbiota on Notch signalling transduction. For example, infection with *Listeria monocytogenes* promotes the differentiation of intestinal stem cells into goblet cells by inhibiting the Notch1/Hes1 pathway, which is one of the causes of diarrhoea (110). *Desulfovibrio vulgaris* pathologically increases in cases of UC and activates Notch1 signalling transduction through a pathway independent of TLR4 (111). Flagellin promotes Notch1 signalling transduction and induces IL-6 production through NF-κB and RBP-Jκ mediation (112). *Lactobacillus acidophilus* can inhibit the Notch1 signalling pathway, promote intestinal mucosal repair (113), and improve the reduction of goblet cells in cases of Salmonella infection-induced UC (114). *Helicobacter pylori* inhibits the Notch signalling pathway by promoting nucleotide-binding leucine-rich repeat-containing receptor 12 expression, thereby inhibiting the expression of Monocyte chemoattractant protein-1 and Macrophage inflammatory protein-1α in intestinal epithelial cells and thus attenuating Dextran sulfate sodium (DSS)-induced UC (115).

The gut microbiota regulates the Notch signalling pathway and affects UC, and probiotic therapy has exhibited good outcomes (116); however, the corresponding relationship between the microbiota and the effects remains unclear, and the specific mechanism is quite vague. Therefore, we believe that studying the intervention of gut microbiota metabolites in the Notch signalling pathway and their effects on UC is important, as dietary control and nutritional therapy have shown their value (117). The composition of gut microbiota metabolites is relatively simple, making it easy to find the corresponding relationship between substances and effects, thereby guiding clinical treatment.

**Figure 9:**
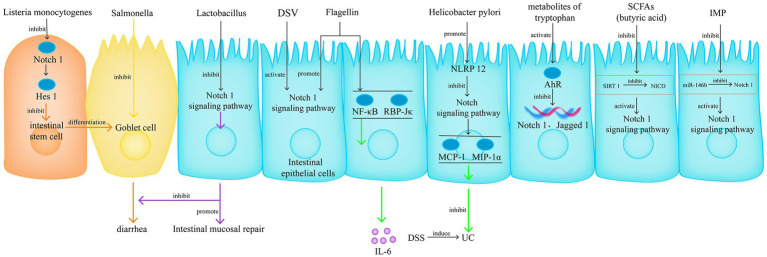
The interaction between the Notch signalling pathway and gut microbiota is an important research direction for regulating host life activities and immune responses. Currently, there are two research approaches, one is focuses on the influence of gut microbiota on Notch signalling transduction, another one is the intervention of gut microbiota metabolites in the Notch signalling pathway and their effects on UC.

Short-chain fatty acids are fermentation products of indigestible carbohydrates produced by symbiotic bacteria, including propionic and butyric acids. Butyric acid can inhibit Sirtuin 1-mediated NICD deacetylation and induce activation of the Notch1 signalling in intestinal epithelial cells (118). In addition, histidine is an amino acid widely present in meat, and its main harmful microbial metabolite is Inosine monophosphate (IMP). IMP disrupts the inhibition of Notch1 by miR-146b, resulting in increased Notch1 signalling, affecting goblet cell proliferation, disrupting the intestinal barrier, and inducing intestinal inflammation (119). Tryptophan (TRP) is an essential amino acid that can only be obtained from a high-protein diet. The gut microbiota can metabolise TRP through the TRP-indole pathway, some of the metabolites can act as ligands for the AhR, and the AhR signal can inhibit the expression of downstream Notch1 and Jagged1 genes (Figure 9) (120).

## 4 Conclusions and future prospects

The Notch signalling pathway associated with UC pathogenesis has distinctive characteristics, demonstrating good regulatory effects on chemical and physical barriers. However, research on UC has mainly focused on regulating immunity, anti-inflammatory effects, and antioxidant stress. Therefore, the study of the Notch signalling pathway suggests the possibility of understanding UC pathogenesis from another perspective. We observed the following problems that deserve attention:

1. The gut microbiota plays an important role in the occurrence and development of UC; however, research should not be limited to the microbiota. Modern research has gradually realised the importance of diet for health. Based on a holistic concept, we consider the gut microbiota as part of the human digestive system. Therefore, the impact of its metabolites on UC is worth noting. The gut microbiota metabolites are the intersection of the gut microbiota and diet. The study of gut microbiota metabolites can explain the combined effect of the two most concerning factors in cases of UC.
2. The Notch signalling pathway affects the repair of intestinal mucosal barriers, especially the chemical and physical barriers. Efficient and precise regulation of the Notch signalling pathway is of great significance in reducing the entry of pathogenic antigens into tissues, achieving anti-inflammatory treatment from another perspective, which differs from immunosuppression, has important theoretical and practical value in addressing the existing limitations of UC treatment, and is opening up new avenues for UC treatment.
3. Animal models are fundamental tools for studying the pathogenesis of diseases. However, no unified modelling method exists for UC animal models. There are various models induced by chemical reagents such as DSS and Trinitrobenzene sulfonic acid, leading to contrasting conclusions, especially in acute/chronic UC models, in which the regulatory effects of drugs on the Notch signalling pathway show almost opposite significant differences (121). Therefore, a unified model that aligns with the pathological mechanism of UC is required.
4. γ-secretase inhibitors are Notch inhibitors, which are potential tools for studying UC and potential drugs for treating UC. However, before conducting extensive research based on γ-secretase inhibitors, some key issues, such as the specificity of target cells, need to be addressed. At this point, Nanoparticles drug delivery system has advantages such as small size, low immunogenicity, diverse surface modifications, and targeting ability. The combination with gamma secretase inhibitors will be a valuable research direction.
5. Through studies involving single and combined gene knockout and overexpression, the different functions of different components in the Notch signalling pathway have been partially revealed. However, the effects of the Notch signalling pathway are influenced by the environment, indicating that Notch signalling can trigger different responses in different cell types at different time points. Further research is needed to understand the overall role of the Notch signalling pathway in UC pathogenesis and how it is regulated.
6. Intestinal stem cells expressing LGR5^+^ are significantly regulated by the Notch signalling and Wnt/β-catenin signalling pathways, which both maintain the function of intestinal stem cells expressing LGR5^+^. In-depth research on these cells based on the Notch signalling pathway may become a new target for UC treatment.
7. Due to the very complex impact of Notch signalling in the intestinal mucosal barrier, any potential therapies aimed at modulating the Notch signalling pathway must be cell-targeted approaches that will preclude any undesired bystander or off-target effects.

In conclusion, our further in-depth research has shown the potentially unique role of the Notch signalling pathway in UC pathogenesis. We hope to provide new directions and ideas for the research and treatment of UC.

## Author contributions

HN: Conceptualization, Writing – original draft. JL: Writing – review & editing. JT: Writing – original draft. MY: Writing – original draft. XL: Conceptualization, Writing – review & editing.
